# Angiosarcoma heterogeneity and potential therapeutic vulnerability to immune checkpoint blockade: insights from genomic sequencing

**DOI:** 10.1186/s13073-020-00753-2

**Published:** 2020-07-09

**Authors:** Amélie Boichard, Michael J. Wagner, Razelle Kurzrock

**Affiliations:** 1 Center for Personalized Cancer Therapy, University of California, Moores Cancer Center, La Jolla, CA 92093 USA; 2 Divison of Medical Oncology, University of Washington and Clinical Research Division, Fred Hutchinson Cancer Research Center, Seattle, WA USA

**Keywords:** Angiosarcoma, Mutagenesis, Personalized therapy, Immunotherapy

## Abstract

**Background:**

Angiosarcoma is an aggressive tumor. Recent case series describe exceptional responses to checkpoint blockade in this disease.

**Methods:**

Herein, we explored the genomic correlates of 48 angiosarcomas from the Angiosarcoma Project (12,499 variants analyzed in 6603 genes; whole-exome sequencing) versus 10,106 pan-cancer tumors in The Cancer Genome Atlas including 235 sarcomas but no angiosarcoma.

**Results:**

At the molecular level, angiosarcomas were heterogeneous. Those located in the face and scalp presented high tumor mutation burden, missense amino acid variations biased towards more hydrophobic (and therefore more immunogenic) peptides, and ultra-violet mutational signature.

**Conclusions:**

Angiosarcoma molecular features are similar to those observed in melanoma and other skin tumors and may explain comparable immunotherapy sensitivity of these tumor types.

## Background

Angiosarcoma is a highly aggressive cancer of endothelial cells. Patients diagnosed with angiosarcoma lesions have poor survival, with a 5-year survival estimated around 20–35%. This rate is possibly explained by an advanced stage at presentation, lack of complete excision, and high prevalence of recurrence and distant metastases [1]. Primary sites for angiosarcoma demonstrate distinct survival rates: soft-tissue angiosarcomas and breast angiosarcomas present the best 5-year overall survival rates of 74% and 51%, respectively, while visceral angiosarcomas of the liver and heart are almost universally fatal within 5 years from diagnosis [2]. Cutaneous angiosarcomas (representing up to 60% of all angiosarcomas) have a relatively good prognosis compared with primary tumors of other sites, with a 5-year overall survival of 43% [2]. Interestingly, angiosarcomas of the scalp seem to be more aggressive than tumors of the face, with a 5-year overall survival of 9% compared to 23% for other facial lesions [3].

Effective therapeutic options available for patients diagnosed with angiosarcoma can carry high morbidity and often do not lead to durable response. Primary treatment for localized disease includes surgery and radiation, with negative surgical margins difficult to obtain due to the infiltrative growth pattern of the disease and radiation therapy limited in cases of radiation-induced tumors. Systemic chemotherapy, predominantly using anthracyclines and taxanes, is the cornerstone of treatment for metastatic and advanced disease [1, 4]. Recently, Florou et al. reported in a small case series exceptional response rates for checkpoint blockade immune therapy. In 7 patients treated with checkpoint inhibitors, 5 (71%) responded positively [5]. This cohort included 5 head and neck (4 responders) and 2 breast angiosarcomas (1 responder). Before this report, anecdotal cases of response to checkpoint blockade have been described (Additional file 1: Table S1) [6–8]. Since many sarcomas have low rates of response to immunotherapy, we explored the genomic correlates of angiosarcomas to determine if there are underlying features that might predict checkpoint blockade sensitivity.

## Methods

We analyzed 10,106 pan-cancer tumor samples from The Cancer Genome Atlas (TCGA https://www.cancer.gov/about-nci/organization/ccg/research/structural-genomics/tcga) including 235 samples with sarcoma [9] and 441 samples with melanoma [10]. TCGA does not include angiosarcomas. We also evaluated molecular data from 48 tumor samples corresponding to 36 unique patients that were downloaded from the Angiosarcoma Project (https://ascproject.org/) [11]. Whole-exome sequencing extraction resulted in a total of 12,499 variants analyzed (located in 6603 different genes) from the angiosarcoma samples. Investigation of mutation signatures (30 different etiologies) was performed using the Mutational Signature in Cancer (MuSiCa) [12] tool available online at http://bioinfo.ciberehd.org:3838/MuSiCa/.

For both TCGA and the Angiosarcoma Project cohorts, genomic variation descriptions were obtained by whole-exome sequencing (WES) and inferred using the MutSig2CV algorithm. TCGA data version from January 28, 2016, was used. To compare both cohorts, the total number of mutations observed in a given sample was considered. The tumor mutation burden for the Angiosarcoma Project samples was calculated by dividing the total number of mutations in a given sample by the length of genomic regions captured by WES. All data generated for this study is available in Additional file 2: Tables S2–3.

## Results

We analyzed a total of 12,499 point mutations identified from a collection of 48 angiosarcoma tumor samples originating from 36 unique patients (collection publicly available from the Angiosarcoma Project https://ascproject.org/), as well as the tumor mutation burden estimates of 10,106 pan-cancer tumor samples from TCGA (The Cancer Genome Atlas https://www.cancer.gov/about-nci/organization/ccg/research/structural-genomics/tcga) including 235 samples with sarcoma (none of which were angiosarcomas) and 441 samples with melanoma.

**Fig. 1:**
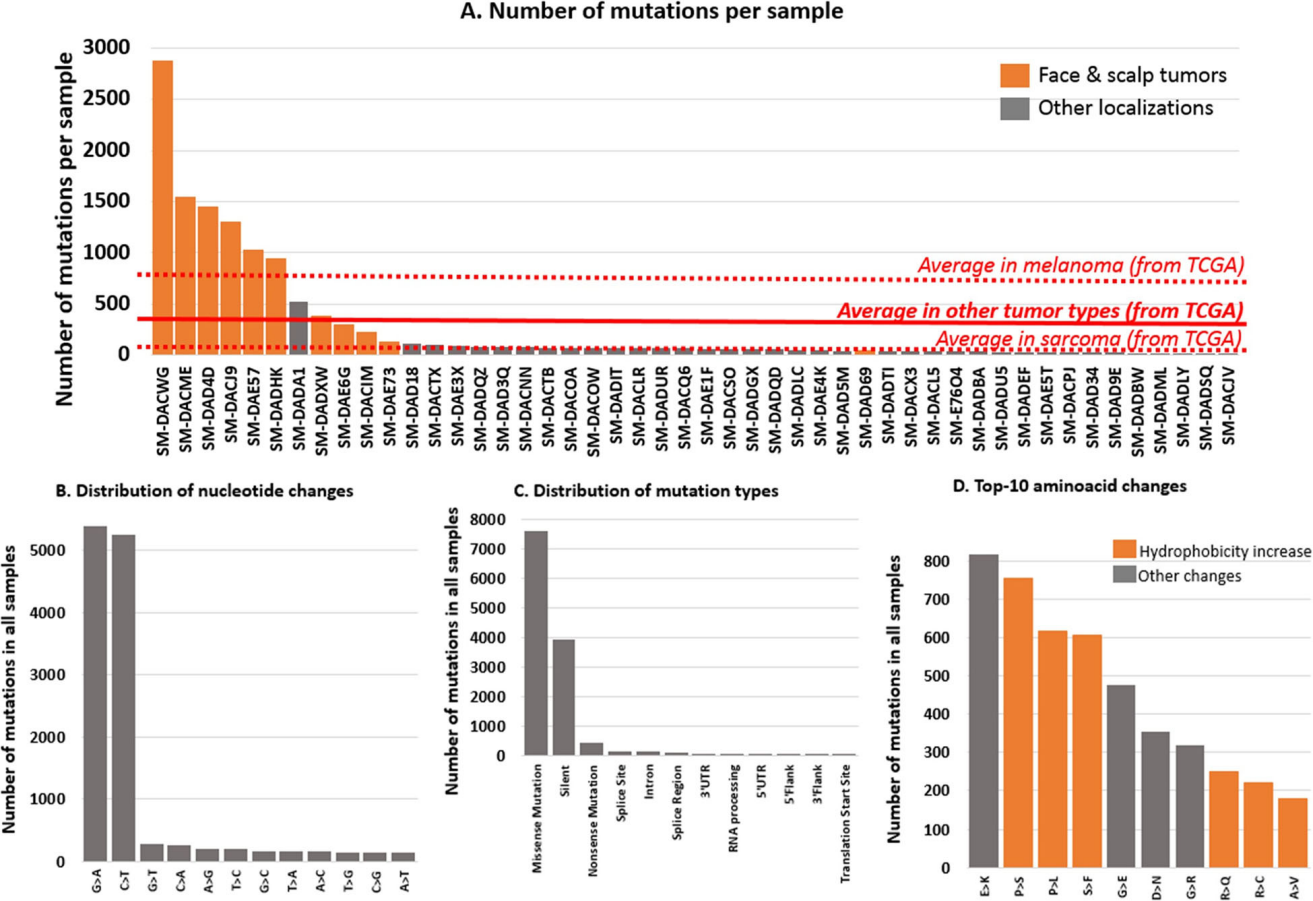
Distribution of mutations identified in the cohort of 48 angiosarcoma samples (The Angiosarcoma Project—https://ascproject.org/). **a** Number of mutations per sample in the Angiosarcoma Project collection of 48 tumors (mean = 260 mutations/sample); the mean in the subgroup of face and scalp angiosarcoma is 925 mutations/sample (*N* = 11 samples). In comparison, the mean number of mutations per sample in The Cancer Genome Atlas (TCGA) pan-cancer cohort is 212 mutations/sample (*N* = 10,106 samples; *p* value = 0.019), in the sarcoma cohort is 74 mutations/sample (*N* = 235 samples; *p* value = 0.008), in the melanoma cohort is 742 mutations/sample (*N* = 441 samples; *p* value = 0.496), and in the Angiosarcoma Project non-face/non-scalp subgroup is 63 mutations/sample (*N* = 37 samples; *p* value = 0.007). **b**–**d** Classification of mutations observed in 48 angiosarcoma samples by physicochemical properties (nucleotide, mutation types, and amino acid changes). A total of 12,499 variants were analyzed. Hydrophobicity increase reflects greater immunogenicity, as described by [13–15]

Angiosarcoma tumors presented an average of 260 mutations per sample (from 8 to 2868 mutations per sample). Higher mutation burden was observed in angiosarcomas of the face and scalp, presenting an average of 925 mutations per sample (*N* = 11 samples) (Fig. 1a). In comparison to the face and scalp angiosarcomas, the typical mutation burden in the non-face/non-scalp angiosarcoma subgroup was 63 mutations per sample (*N* = 37 samples; *p* value = 0.007); 212 mutations per sample in the TCGA pan-cancer cohort (*N* = 10,106 samples; *p* value = 0.019); 74 mutations per sample in the TCGA sarcoma cohort (*N* = 235 non-angiosarcoma samples; *p* value = 0.008); and 742 mutations per sample in the TCGA melanoma cohort (*N* = 441 samples; *p* value = 0.496) (Fig. 1a).

The main nucleotide changes were G>A and C>T transitions for 85% of the variants in the 48 angiosarcomas (*N* = 5392/12,499 and 5250/12,499 variants, respectively) (Fig. 1b). These variants resulted in missense (*N* = 7597/12,499 variants; 61%) or silent (*N* = 3931/12,499 variants; 31%) protein mutations (Fig. 1c). Missense amino acid changes were biased towards more hydrophobic residues in 57% of the mutations (*N* = 4326/7597 variants); such changes represented 6 out of the 10 top modifications (Fig. 1d). The mean hydrophobicity was significantly higher after missense mutation in comparison to the human reference protein sequences (paired *T* test *p* value < 0.0001; mean hydrophobicity change [95% confidence interval] = + 0.9240 [(+ 0.8555) − (+ 0.9924)] (as calculated by using the Kyte-Doolittle hydrophobicity index where hydrophobic residues present an index greater than zero).

**Fig. 2:**
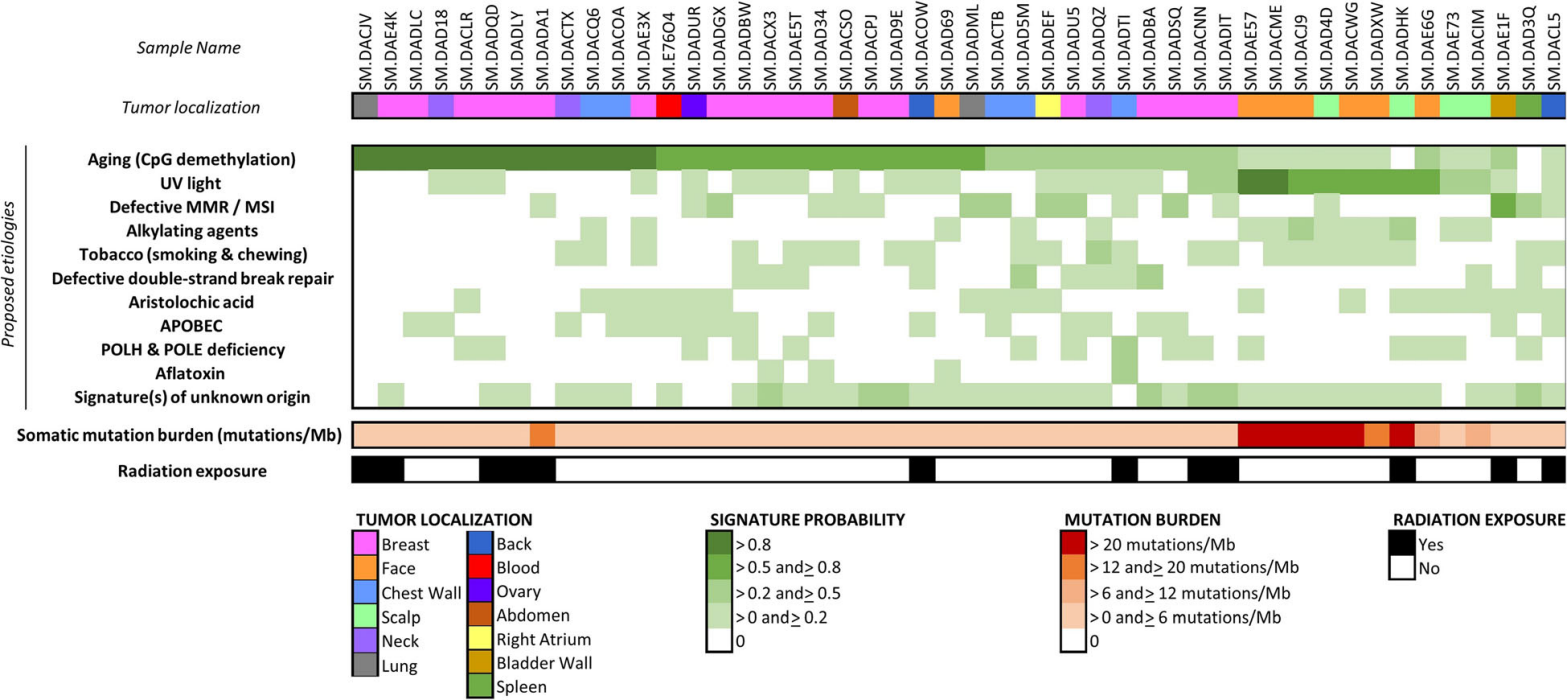
Mutational signature analysis of primary angiosarcoma (*N* = 48 samples, 12,499 single nucleotide variants analyzed). The figure shows that face and scalp angiosarcomas often have high or intermediate mutation burden and UV light signature whereas other angiosarcomas have mostly lower mutation burden and aging signatures. Each etiology was studied individually. The signature probability was calculated using the entire set of variants in each sample. The probability (see green spectrum) is given as a number between 0 and 1: 0 corresponds to the lowest probability that the causative event occurred in the tumor and 1 corresponds to the highest probability that the causative event occurred in the tumor. Aging (as demonstrated by spontaneous demethylation of CpG islands), ultra-violet (UV) light exposure, and defective mismatch repair (MMR) are the top-3 etiologies proposed. Abbreviations: APOBEC, apolipoprotein B mRNA editing enzyme, catalytic polypeptide-like; Mb, megabase; MMR, mismatch repair; MSI, microsatellite instability; POLE, polymerase epsilon; POLH, polymerase eta; UV, ultra-violet

The analysis of putative etiology of genomic point mutations observed in angiosarcoma (study of mutational signatures) revealed that most angiosarcoma samples present a high probability of variations caused by the spontaneous demethylation of CpG islands. Face and scalp angiosarcomas were more specifically impacted by mutations previously attributed to ultra-violet (UV) radiation exposure, and rare visceral angiosarcomas presented high probability of mismatch repair deficiency (Fig. 2).

## Discussion

Angiosarcoma samples are not specifically prone to hypermutativity processes. However, some samples showed a high number of variants (Fig. 1a). Interestingly, high tumor mutation burden has already been described as an independent immunotherapy predictive biomarker in other tumor types such as melanoma, basal cell carcinoma, and squamous cell carcinoma [13, 14, 16–18]. Although no checkpoint blockade outcome data was available at the time of the analysis for the angiosarcoma cohort, we can hypothesize that the high number of mutations observed in face and scalp angiosarcoma may be a driver of favorable outcomes to checkpoint inhibitor therapy in this disease. This hypothesis is consistent with published anecdotal data (Additional file 1: Table S1) demonstrating that response to anti-PD1 agents is seen mostly in face and scalp angiosarcomas. Further clinical investigation is warranted.

Genomic variants observed in angiosarcoma are biased towards G>A and C>T transitions, and missense variants, when expressed, are more likely to results in more hydrophobic amino acid residues (Fig. 1b–d). Peptide hydrophobicity is known to correlate with increased antigenicity and immunogenicity [19, 20], and therefore, the characteristics of the missense mutations observed in angiosarcoma samples may confer a specific immune environment and immunotherapy responsiveness to this tumor type.

The observation of specific nucleotide changes could be explained by specific causative events in angiosarcoma. For this reason, we performed an analysis of mutational signatures of tumor samples, as described by Alexandrov et al. [21]. Angiosarcoma samples present a high probability of genomic variations caused by the spontaneous demethylation of CpG island (a phenomenon related to the aging process) except for face and scalp angiosarcomas that seem to be specifically impacted by ultra-violet (UV) light (Fig. 2). The UV light signature is found in melanoma and other skin tumors and may, therefore, be another similarity with tumor types known to respond to PD-1/PD-L1 blockade. The high prevalence of UV-induced genomic variants appears to be a specific hallmark of face and neck angiosarcoma, as recently described by the patient-partnered Angiosarcoma Project initiators [11].

To note, some non-face/non-scalp angiosarcomas also present a mismatch repair (MMR) defect signature, another predictive marker for immunotherapy highly correlated with elevated tumor mutation burden [22] (Fig. 2). However, in published reports, angiosarcomas and other tumors presenting with low tumor mutation burden also responded to immunotherapies (Additional file 1: Table S1). Therefore, a high prevalence of mutations in tumors does not always predict for response to checkpoint blockade; other predictive biomarkers may be important.

No PD-L1 protein evaluation was available from the Angiosarcoma Project, and we could not assess for PD-L1 expression in these samples. Previously published reports show variable levels of PD-L1 expression in angiosarcomas. A study of 24 primary angiosarcomas revealed PD-L1 expression in about 66% of samples; PD-L1-positive samples were mainly observed in bone (4/4—100%), soft tissue (4/5—80%), skin (3/4—75%), breast (4/7—57%), and visceral (1/4—25%) localizations [23]. Other reports described lower rates of PD-L1 tumor expression, estimated between 14% and 19% [24, 25]. It is also known that 60% of canine hemangiosarcomas, a tumor entity sharing high molecular resemblance with human angiosarcoma [15], express PD-L1 [26]. Both human and veterinary studies may highlight an important specificity of angiosarcoma tumors and explain, at least in part, their exquisite sensitivity to checkpoint inhibitors.

## Conclusions

In this study, we aimed to describe potential molecular biomarkers explaining the initial reports of angiosarcoma sensitivity to checkpoint inhibitors. Our analysis, based on a public dataset derived from 48 tumor samples available from the Angiosarcoma Project (https://ascproject.org/), indicates that, at the genomic level, angiosarcomas are heterogeneous. Of interest, facial and scalp angiosarcomas harbor high tumor mutation burden and ultraviolet (UV) light mutational signature [21], both features that have been implicated in checkpoint blockade responsiveness [16]; the mutations in these tumors also create an amino acid shift towards hydrophobicity, which is associated with immunogenic neo-peptides.

Our study has several limitations including the lack of clinical correlative data, lack of PD-L1 expression data, and the restricted number of samples. Future investigations will be needed to address these issues. Even so, the description of the mutational landscape of angiosarcomas may help to elaborate on specific therapeutic approaches. Interestingly, there are now clinical trials assessing the efficacy of immunotherapeutic agents in patients with angiosarcoma. These include a phase II clinical trial of dual PD-1 and CTLA-4 inhibition with nivolumab and ipilimumab (DART trial, NCT02834013), a similar phase II trial of dual PD-L1 and CTLA-4 inhibition with durvalumab and tremelimumab (NCT028115995), and a combination of the PD-L1 inhibitor avelumab with the standard chemotherapeutic agent paclitaxel (NCT03512834). These trials should further elucidate the activity and molecular correlates of checkpoint blockade in this disease and whether mutational burden/UV signature determines response, as well as predominant sites (such as scalp and face angiosarcomas) that are most likely to be successfully treated with immunotherapy.

## Supplementary information

**Additional file 1: Table S1.** Reports of angiosarcoma response to immunotherapy by checkpoint PD-1/PD-L1 blockade previously published.

**Additional file 2: Table S2.** Genomic variants found by whole-exome sequencing in 48 angiosarcoma samples from the Angiosarcoma Project. **Table S3.** Description of 48 angiosarcoma samples from the Angiosarcoma Project and corresponding mutational signature probabilities.

## Data Availability

All data used in this study originating from the Angiosarcoma Project [11] and The Cancer Genome Atlas sarcoma [9] and melanoma [10] cohorts (TCGA https://www.cancer.gov/about-nci/organization/ccg/research/structural-genomics/tcga) is publicly available through the cBioPortal website (https://www.cbioportal.org/). All results generated for this study are available in Additional file 2: Tables S2–3

## Abbreviations

CTLA-4: Cytotoxic T-lymphocyte-associated protein 4
MMR: Mismatch repair
MuSiCa: Mutational Signature in Cancer
MutSig2CV: Mutation Significance version 2CV
N: Number
PD1: Programmed cell death 1
PD-L1: Programmed death-ligand 1
TCGA: The Cancer Genome Atlas
UV: Ultra-violet
WES: Whole-exome sequencing
