# Supplementary material for: Angiosarcoma heterogeneity and potential therapeutic vulnerability to immune checkpoint blockade: insights from genomic sequencing
Source: Genome Med. 2020 Jul 9;12:61. doi: 10.1186/s13073-020-00753-2 (PMC7350570; doi:10.1186/s13073-020-00753-2)
Supplement: Supplementary file 1 — Additional file 1: Table S1. Reports of angiosarcoma response to immunotherapy by checkpoint PD-1/PD-L1 blockade previously published. [file 13073_2020_753_MOESM1_ESM.docx]

**Table S1:** Angiosarcoma response to immunotherapy by checkpoint PD-1/PD-L1 blockade previously reported in the literature.

| **Year** | **Number of cases** | **Tumor description** | **Tumor molecular profile** | **Treatment** | **Response** | **Comment** | **Ref.** |
| --- | --- | --- | --- | --- | --- | --- | --- |
| 2017 | 1 | Cutaneous face,  hepatic recurrence | PD-L1 positive  (SP142 antibody, >5% cells) | Pembrolizumab | PR |  | ^6^ |
| 2018 | 1 | Primary hepatic | PD-L1 negative  VEGFR2 positive  No known mutations  (295-gene NGS panel) | PD-1 inhibitor + pazopanib + RAK cells | PR |  | ^7^ |
| 2018 | 1 | Cutaneous retro-auricular with lymphatic recurrence | PD-L1 positive  (22C3 and 28-8 antibodies, 10% cells)  *TP53* mutation  (17-gene NGS panel) | Pembrolizumab | CR |  | ^8^ |
| 2019 | 7 | Cutaneous face (n=2)  Cutaneous scalp (n=3)  Primary breast (n=1)  Radiation-induced breast (n=1)  (all advanced disease) | **Patient with CR:**  PD-L1 positive  *NBPF10*, *NBPF15*, *ZNF678*, *VPS8*, *PCLO* and *ABCB1* mutations  TMB low  (NGS whole exome)  **Patient with cutaneous PR:**  *CDKN2A/B, CHEK2, DNMT3A, FANCD2, MLL3, and TP53* mutations  TMB intermediate  (NGS FoundationOne)  **Patient with cutaneous PR:**  *CRKL, DNMT3A, MAPK1, SF3B1 and ZRSR2* mutations  TMB intermediate  (NGS FoundationOne) | Pembrolizumab (n=4)  Pembrolizumab + axitinib (n=1)  CTLA-4 inhibitor (n=2) | Objective responses in 5 of 7 patients (includes 1 CR) | Response was observed in 2 advanced facial cutaneous (includes a CR), 2 scalp cutaneous and 1 radiation-induced breast angiosarcomas. | ^5^ |

**Abbreviations:** CR = complete remission; CTLA-4 = cytotoxic T-lymphocyte-associated protein 4; NGS = next-generation sequencing; PD-1 = Programmed cell death 1; PD-L1 = Programmed death ligand 1; PR = partial remission; TMB = tumor mutation burden.
